# Computerized Exercises to Promote Transfer of Cognitive Skills to Everyday Life

**DOI:** 10.3389/fpsyt.2016.00056

**Published:** 2016-04-15

**Authors:** Pascal Vianin

**Affiliations:** ^1^Département de Psychiatrie, Centre Hospitalier Universitaire Vaudois (DP-CHUV), Lausanne, Switzerland

**Keywords:** cognitive remediation, computer, metacognition, procedural knowledge, transfer, RECOS therapy

## Abstract

In recent years, computerized and non-computerized cognitive remediation programs have been designed for both individual and group settings. We believe, however, that a common misconception lies in considering the efficiency of a cognitive remediation therapy as resulting from the sole use of a computer. This omits that metacognitive skills need also to be trained throughout the remediation phase. RECOS is a theory-based therapeutic approach designed to promote the transfer of cognitive skills to functional improvements. It involves working with one person at a time using both paper/pencil tasks and a set of interactive computer exercises. Paper/pencil exercises are used to promote problem-solving techniques and to help patients to find appropriate suitable strategies. During the following computerized 1-h session, therapists guide participants to the procedural dimension of the action, which refers to knowledge about doing things and relies on retrospective introspection. We assume that each patient has a rich and underestimated procedural knowledge he/she is not aware of. By providing complex and interactive environments, computerized exercises are recommended to bring this knowledge to light. When strategies used by the participant become conscious, conditional knowledge determines when and why to use them in real-life situations.

## Introduction

Individuals who suffer from mental illness including schizophrenia, bipolar disorder, or depressive disorder often experience cognitive deficits. Attention, processing speed, executive functions, or memory deficits may lead to severe disability in everyday life. Therefore, improving cognitive abilities is essential for the well being of the patients. During the last decades, cognitive remediation was developed first for traumatic brain injury and later for mental disorders.

Cognitive remediation programs alone have shown moderate improvements on cognitive and functional outcomes. With the objective of improving the beneficial effects of cognitive training, meta-analyses aimed to determine what factors moderate outcome to cognitive remediation therapy. Most of them identified treatment variables. For example, they showed that combining cognitive remediation with a psychiatric rehabilitation program (1, 2) or linking the remediation stage with real-life situations (3) increased the effectiveness of these programs.

Several studies show, however, that the way cognitive performance is improved by cognitive remediation therapy is different according to the patient’s neuropsychological profile (4, 5). For that reason, we believe that many programs lack specificity, the patients being generally confronted with similar executive, attention, or memory exercises. Given the heterogeneity of the cognitive profiles of individuals with schizophrenia, remediation should be adjusted to each individual’s needs. Therefore, the theoretical foundation of RECOS emphasizes personalization of the treatment by providing specific and targeted cognitive training modules.

Several cognitive remediation programs aim at improving neurocognitive functions (e.g., CRT, NEAR, and RECOS), whereas other interventions target social cognition (e.g., TAR, SCIT, and TomRemed) in severe mental diseases. A further distinction can be made between cognitive remediation techniques relying on paper and pencil support and those that are computer assisted. In a recent review, Grynszpan et al. (6) summarize the advantages of computer-assisted cognitive remediation: unlimited training possibilities, automatic reinforcement, multisensory presentation, objective recording of performance, standardized training tasks, intrinsic motivation, etc.

However, we do not believe that the sole use of a computer may predict the success of the cognitive remediation therapy. We assume that metacognitive skills are more important to consider than the media used for improving cognition in real-world settings. Improving knowledge about his/her own cognition and the way strategies can be used for everyday functioning seems to be essential for recovery. In our view, cognitive remediation therapy should be based on a functional and metacognitive approach. By providing ecological and multisensory strategies, computer activities offer opportunities for improving not only cognitive functioning but also metacognitive skills. The present paper aims to show how computerized exercises used in RECOS therapy should promote the transfer of cognitive skills to functional improvements.

## Recos Therapy

RECOS – *COgnitive REmediation for Schizophrenia or other related disorders* – is an individualized cognitive rehabilitation treatment to take into account the cognitive heterogeneity characterizing this disorder (7). It includes training in six of the most highly altered areas of cognition: verbal memory, visuospatial attention and memory, working memory, selective attention, executive functions, and processing speed. Remediation phase is preceded by a comprehensive neuropsychological assessment. Before beginning cognitive training, functional consequences of cognitive troubles are evaluated with each patient using qualitative criteria. To facilitate generalization of cognitive gains to everyday life, interventions are aimed at concrete goals defined according to the patients’ difficulties and discussed regularly throughout the therapy. At the end of the remediation stage, participants are re-evaluated by using a similar neuropsychological battery. In order to explore the effectiveness of the RECOS therapy, a preliminary study showed that effect sizes were more important for each cognitive function when they have been trained rather than untrained (8), suggesting that matching treatment to each individual’s level of cognitive functioning is essential for cognitive enhancement. In a randomized study, Franck et al. (9) found that participants to RECOS training improved not only their cognitive performance but also their clinical and social functioning.

RECOS exercises were designed by Scientific Brain Training Company (SBT, Villeurbanne, France – http://www.sbt.fr/) and adapted by our team for specific use in schizophrenia. They were designed to be engaging and similar to everyday situations in order to facilitate generalization to everyday life skills and to enhance motivation. The remediation phase includes 28 1-h twice-weekly sessions with the therapist and 14 sessions completed at home (42 h in total). Each week, participants take part successively in the paper/pencil and computer sessions with the therapist and are asked to do exercises at home.

The first session of the week is dedicated to paper/pencil exercises. Each exercise is chosen according to the cognitive abilities solicited by the individual objective of the participant. Problem-solving strategies are mainly employed during paper/pencil sessions. The problem-solving cycle begins by identifying and defining the problem. During the next step, participants are asked to suggest a large number of strategies to solve the task. After a discussion with the therapist, the apparently effective solutions are then selected. After this selection, it is important to evaluate the results to determine the best possible solution to the problem. At the end of the paper/pencil session, the following home work is decided, aiming to transfer the learned solution in real-life situations. One week later, the new paper/pencil session begins with a discussion of the exercises made at home. During the whole cognitive remediation program, therapists have to make sure that participants become more and more efficient to transfer the acquired skills to their daily life.

The second weekly session with the therapist is dedicated to computer exercises. Each exercise depicts a daily life situation, such as preparing a cocktail or the storage of supplies. The first two or three levels of each exercise are rather easy, so that the performance is positively reinforced right from the start. To move up a level, it is necessary to achieve a 100% score at this same exercise. Thus, exercises are adapted to the patients’ abilities and help maintain patients’ motivation. Moreover, results are recorded and automated feedback allows patients to observe their progression permanently from the beginning of the program. It appears that with computer-assisted learning, participants become more attentive to the task. As computers provide multisensory stimulation and personalization of activities, they allow the therapist to observe the participants in a quite similar way they would be in a real environment. Unexpected and non-conscious strategies intervene during the computer exercises, giving access to procedural knowledge.

The role of the therapist is quite different in paper/pencil as compared to computer exercises. The therapist is in front of the participant during the paper/pencil whole session, the attention of both being devoted to the exercise. In order to stimulate problem-solving strategies, the therapist encourages the participant to generate and experiment different kinds of solutions. On the contrary, the participant is fully focused on the exercise during the computer session. The therapist is sited behind him/her and does not appear in his/her field of vision. The therapist precisely observes the way the participant is solving the problem (by counting with his/her fingers, looking at the bottom left corner of the screen, moving impulsively his/her mouse, etc.). All this information is useful for the therapist to identify the procedural knowledge of the participant. *Explicitation* interview will complete this information after the end of the exercise (see below).

## Training Metacognitive Skills

Although the concept of *metacognition* is frequently used in literature on cognitive remediation, it has been frequently neglected as an object of treatment. During training sessions, metacognitive skills are essential because they enable participants to manage their cognitive skills better and to identify weaknesses that can be corrected by developing new strategies. Three kinds of metacognitive knowledge are targeted during the phase of training.

### Metacognitive Knowledge

Metacognitive knowledge refers to knowledge not only about one’s own cognitive skills but also about cognitive functions in general, and the way cognitive skills may help participants in their daily life. During the first meeting with their therapist, they indicate the main cognitive difficulties they have recently identified. After the following neurocognitive assessment, standardized results are discussed with them. Confronting these results with their initial complaints generally allows them to become aware not only of their difficulties but also of their resources. Throughout the program, participants are then encouraged to identify cognitive processes engaged in their activities and exercises. Moreover, a whole paper/pencil session is dedicated to psychoeducation on cognitive functions at the beginning of the therapy. During this 1-h discussion, the therapist explains the cognitive functions treated by RECOS therapy, the importance to improve functional outcome, the impact of specific impairments in different areas of the daily life, etc.

### Procedural Knowledge

Procedural knowledge refers to knowledge about doing things. It is typically exercised in the accomplishment of a task. Piaget (10) underlines that the action is a non-conscious and autonomous knowledge. Indeed, participants to RECOS therapy often learn procedural knowledge without even being aware that they are learning. In order to bring this knowledge to light, Vermersch (11) developed the *explicitation interview*, which relies on the participant’s retrospective introspection. It draws on Piaget’s theory of how experience is processed into reflection. It requires that the participant is guided toward the verbalization of the lived experience (12). To accomplish this, interviewees enter a state of evocation, so that they are “reliving” the activity. The interviewer should establish a state of evocation in the participant by focusing on procedural dimension of the action.

During RECOS therapy, procedural knowledge is best identified during computer exercises (13). Because of computer ecological and multisensory presentation of tasks, participants feel generally present in the virtual environment. While paper/pencil sessions request a collaborative relation with the therapist, computer sessions allow to evaluate the participant “in action.” *Explicitation* interview may be introduced by the therapist after the participant ends a new exercise. It begins with what is called *a contract of evocation*: “What I suggest to you, if you agree, is to take your time, allowing a moment to explain how you realize this exercise.” The therapist encourages participants to evoke a particular episode of the computerized exercise he/she has just realized: “Please tell me what you did as soon as the ingredients of the cocktail appeared on the screen, until the moment you gave your answers by clicking on the validation button.” Different cues, such as the tone of the voice, the words used, or the gaze of the participants, reveal whether they are in evocation or not. It is important that the therapist does not sit directly opposite the participants as this interferes with the fact of becoming aware of the lived experience. In order to be in evocation, the participant should not explain to the therapist the strategies used but has to live the exercise again by verbalizing his/her thoughts when performing it. The interviewer avoids questions such as “why,” which brings on rationalizations. When the participant makes judgments about his/her performance, he/she is invited to come back to the way he/she proceeded to solve the task. Results obtained at the end of the exercise should, therefore, not be considered during the *explicitation* interview.

### Conditional Knowledge

Conditional knowledge refers to knowing when and why to use declarative and procedural knowledge. It allows participants to allocate their resources when using strategies. Conditional knowledge is therefore essential to transfer strategies identified by the *explicitation* interview. Home exercises aim to help participants to transfer these skills to reach the objectives of RECOS therapy. In the case of Sofia (Box 1), homework may consist in using strategies used during the *Heraldry* exercise in the professional environment to remember the work done on the seats of the old cars. The therapist invites her to imagine how such abilities would help her to remember colors and shapes of the seats she worked on last time.

Box 1 **Case report.**

Sofia is a young woman with schizophrenia. She works 2 days a week in a garage. Her job is to change the colors for the seats of old cars, according to the clients’ choice. Because of her deficits in visuospatial memory, she is not able to remember what she has done as soon as she interrupts her work for some minutes or hours. The objective of remediation is to help her to remember shapes, colors, and patterns of the seats she has to fix. For this purpose, the therapist suggests her to solve the exercise *Heraldry*. In the computerized exercise, Sofia is asked to memorize a coat of arms. She will be asked to recreate it with its components when it will have disappeared from the screen.

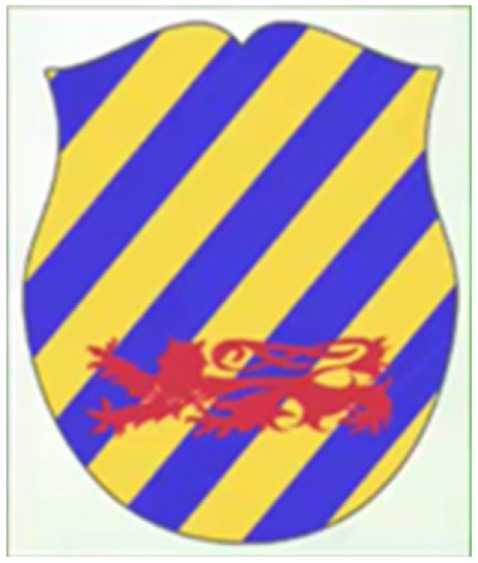

After Sofia has finished this exercise successfully, she is asked whether she agreed to be interviewed about this exercise. After her acceptance, she begins the explicitation interview by describing her successive thoughts.

First I look at the shape of the flag. I observe that the top is like the shape of a mouth whereas the bottom of it is like a tongue (laughs) (…) Then I look at the colors of the lines. I see that they are blue and yellow. I repeat several times in my head ‘yellow, blue, yellow, blue, yellow, …’ (…) and then I observe that the red figure looks like the Agip gas station logo …

The interview lasts several minutes and allows her and the therapist to become aware of the numerous strategies used during the exercise. It is quite remarkable that Sofia generates so many strategies without any suggestion or help from the therapist. Before RECOS therapy, she explained that she suffers from schizophrenia and therefore does not have any strategies to solve her problems of memory!

Note: The actual Case Report introduced in the paper concerns a patient who participated to the RECOS therapy as a patient. I was her therapist, and she did not participate in any study about cognitive remediation. Information has been provided to the patient who agreed to be cited in articles or in a training about cognitive remediation. Her name has been anonymized (Sofia is not her real name). There is no need to submit to an ethical committee for such a case report in our institution.

**Figure 1:**
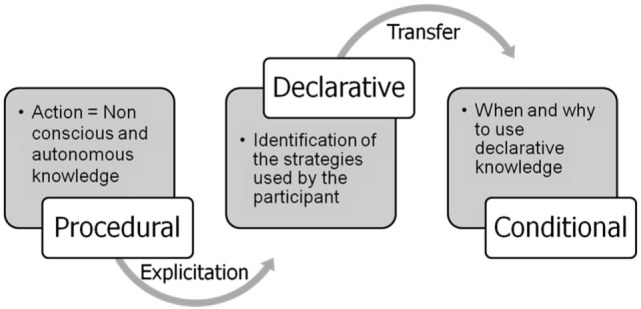
**Participants are asked to evoke strategies used during the exercises**. When strategies used by the participant become conscious, conditional knowledge determines when and why to use them in real-life situations.

These metacognitive skills are key determinants to improve functional abilities of the participants (Figure 1). When procedural knowledge becomes conscious, it becomes declarative knowledge and can be directly and explicitly applied to a problem-solving task. Consciousness of his/her procedural knowledge is therefore fundamental because people do understand that they are using strategies. The role of the participant – with the help of the therapist – is then to identify where and when these strategies become efficient to solve a problem in a specific situation. Conditional knowledge is essential for many patients with schizophrenia because of their lack of cognitive flexibility: they often imagine that a strategy that has worked once is a good strategy. They need to consider the context in which such a strategy may be useful. Improving conditional knowledge is probably the main difficulty RECOS therapists are confronted with.

## Conclusion

We believe that a common misconception is considering the efficiency of a cognitive remediation therapy as resulting from computerized exercises. This omits that metacognitive skills also need to be trained during the whole remediation phase. In our view, efficiency of cognitive remediation therapy depends more on the possibility of the participants to transfer acquired skills during sessions in everyday life than from the type of exercises used in the remediation phase. Our metacognitive approach tries to bridge the gap between basic neurocognition and real-world functioning.

From a neurocognitive point of view, RECOS therapy develops the ability to change personal thought processes since each participant is requested to analyze performance against strategies they have themselves deployed. This work of “metacognitive restructuring” is important because patients tend to think that they have no control over their difficulties. They commonly consider that their failures are simply due to the fact that they suffer from schizophrenia. This sense of “learned helplessness” (14) suggests that the drop in performance is due to repeated failures and the subjective impression that the situation cannot be controlled. On the contrary, the patients must understand that their success is due to the conscious and systematic use of relevant problem-solving strategies and that their failures are due to strategies which are ineffective or inappropriate to the situation.

Vermersch’s use of introspection to give access to procedural learning theory has inspired our conception of cognitive remediation in psychiatry. We are convinced that each patient has a rich and underestimated procedural knowledge that he/she is not aware of. By providing complex environments favorable to induce motivation (15) and because of their non-judgmental nature in case of failure (16), computerized exercises are recommended to bring this knowledge to light.

## Author Contributions

The author confirms being the sole contributor of this work and approved it for publication.

## Conflict of Interest Statement

The author declares that the research was conducted in the absence of any commercial or financial relationships that could be construed as a potential conflict of interest.

## References

[1] McGurk SR Twamley EW Sitzer DI McHugo GJ Mueser KT. A meta-analysis of cognitive remediation in schizophrenia. Am J Psychiatry (2007) 164(12):1791–802. 10.1176/appi.ajp.2007.07060906 18056233 PMC3634703

[2] Wykes T Huddy V Cellard C McGurk SR Czobor P. A meta-analysis of cognitive remediation for schizophrenia: methodology and effect sizes. Am J Psychiatry (2011) 168(5):472–85. 10.1176/appi.ajp.2010.10060855 21406461

[3] Medalia A Saperstein AM. Does cognitive remediation for schizophrenia improve functional outcomes? Curr Opin Psychiatry (2013) 26(2):151–7. 10.1097/YCO.0b013e32835dcbd4 23318663

[4] Kurtz MM Seltzer JC Shagan DS Thime WR Wexler BE. Computer-assisted cognitive remediation in schizophrenia: what is the active ingredient? Schizophr Res (2007) 89:251–60. 10.1016/j.schres.2006.09.001 17070671 PMC2095777

[5] Fiszdon JM Choi J Bryson GJ Bell MD. Impact of intellectual status on response to cognitive task training in patients with schizophrenia. Schizophr Res (2006) 87:261–9. 10.1016/j.schres.2006.04.011 16737798

[6] Grynszpan O Perbal S Pelissolo A Fossati P Jouvent R Dubal S Efficacy and specificity of computer-assisted cognitive remediation in schizophrenia: a meta-analytical study. Psychol Med (2011) 41:163–73. 10.1017/S0033291710000607 20380784

[7] Vianin P La Remediation Cognitive dans la Schizophrénie. Le Programme RECOS. Bruxelles: Mardaga (2013).

[8] Deppen P Sarrasin-Bruchez P Dukes R Pellanda V Vianin P Programme de remédiation cognitive pour patients présentant une schizophrénie ou un trouble associé (Recos): résultats préliminaires. L’Encéphale (2011) 37:314–21. 10.1016/j.encep.2011.02.002 21981893

[9] Franck N Duboc C Sundby C Amado I Wykes T Demily C Specific vs general cognitive remediation for schizophrenia: a multicentre randomised trial. Schizophr Res (2013) 147(1):68–74. 10.1016/j.schres.2013.03.009 23583327

[10] Piaget J Réussir et Comprendre. Paris: PUF (1974).

[11] Vermersch P L’entretien d’explicitation en Formation Continue et Initiale. Paris: ESF (1994).

[12] Vermersch P Describing the practice of introspection. J Conscious Stud (2009) 16(10–12):20–57.

[13] Vianin P Urben S Fornari E Magistretti PJ Marquet P Jaugey L Increased activation in Broca’s area after cognitive remediation in schizophrenia. Psychiatry Res (2014) 221:204–9. 10.1016/j.pscychresns.2014.01.004 24507118

[14] Seligman MEP Learned helplessness. Annu Rev Med (1972) 23(1):407–12. 10.1146/annurev.me.23.020172.002203 4566487

[15] Medalia A Revheim N Casey M. The remediation of problem-solving skills in schizophrenia. Schizophr Bull (2001) 27(2):259–67. 10.1093/oxfordjournals.schbul.a006872 11354593

[16] Bellucci DM Glaberman K Haslam N Computer assisted cognitive rehabilitation reduces negative symptoms in the severely mentally ill. Schizophr Res (2003) 59:225–32. 10.1016/S0920-9964(01)00402-9 12414079

